# Do economic shocks spread randomly?: A topological study of the global contagion network

**DOI:** 10.1371/journal.pone.0238626

**Published:** 2020-09-04

**Authors:** Tamás Sebestyén, Zita Iloskics

**Affiliations:** 1 Faculty of Business and Economics, University of Pécs, Pécs, Hungary; 2 MTA-PTE Innovation and Economic Growth Research Group, Pécs, Hungary; 3 EconNet Research Group, Pécs, Hungary; Yahoo, SPAIN

## Abstract

The spread of economic shocks in an increasingly interconnected global economy has been subject to several studies recently. These studies mostly focus on the synchronization of business cycles among economies and search for the relationship between trade linkages and shock contagion. In contrast to previous studies in the field, this paper focuses on the topological properties of the shock contagion network as measured by pairwise Granger causality between economic output of countries. This topological approach can bring new insights into the dynamics of contagion and the relationship between trade and cycle synchronization while also allows to test the patterns of shock contagion against randomness. Results show that connectedness decreases over the previous decades until the first decade of the 21st century, showing less frequent shock transmission which shades previous results in the field which typically associate increasing trade and globalization with more frequent or unchanged contagion. We find significant non-random topology with respect to transitivity and path lengths, the skewness of the degree distribution and the stability of connections. Estimations show that there is a systematically existing (persistent) contagion path in 16% of all possible connections. However, we do not find significant geographical or development-wise patterns behind the modularity of the contagion network and no significant association is found between economic openness and exposure to shock transmission in either direction.

## Introduction

As the economic system of the world is becoming increasingly globalized and complex [1], more and more studies argue that its analysis requires new approaches integrating specific aspects of complex system research [2, 3]. From a different perspective, network science clearly shows that there is a relationship between the structure of connections among agents and the aggregate performance of the whole networked system [4–6]. It follows from these two arguments that studying economic systems from a network perspective can bring important insights into economic analysis.

Many attempts have recently been made along this line to reveal how economic micro-structure affects aggregate performance. Some of the most influential studies argue that an asymmetric distribution of intersectoral transactions can give rise to heavy tailed aggregate fluctuations [7, 8]. Others show that taking into account the structure of interconnections between market actors can significantly alter predictions from standard economic models through allowing for additional feedback loops in interregional trade flows [9] or welfare loss arising from incomplete supplier-buyer networks [10]. Another vein of literature focuses on financial/inter-bank connections arguing that the structure of these networks are important in estimating the extent of systemic risk arising from the system [11–13].

In addition to these efforts, the structure of the global economy as represented by different flows and connections between countries has also been on the agenda for a while. The study of international trade networks gained special attention along this line due to the easily available data and the importance of these economic connections [14–19]. In addition to topological description, this literature has recently shown that structural changes within the world trade (export) network can be conclusive about the development paths of countries [17, 19]. Another vein within this literature argue that overall instability in the complex global economic system and the 2008 crisis in particular is a result of increased globalization and complexity of the underlying networks [20–22]. Also, interconnected risks in the global economy and cascades on this risk network has been analyzed recently [23, 24]. [18] argue that early signs of the 2008 crisis can be detected in the structure of the world trade network.

Apart from investigating trade networks, attention is also directed towards how national economies affect each other, or differently, to what extent is economic activity synchronized. Most of this research like [25–28] focus on the contemporaneous correlation between some macroeconomic variables, mostly output and/or consumption. More specifically, [26–29] use the growth rate of real Gross Domestic Product and analyze the pairwise correlation between these time series.

While using cross-correlations between time series is common in the financial contagion literature [30], there are some studies claiming for a more sophisticated approach to identify these linkages. [29] and [31] e.g. use Granger causality tests to identify cross-country macroeconomic effects and contagion in sovereign debt risk respectively. Instead of simply relying on the co-movement between several time series, Granger causality is able to detect whether one of the time series is useful in forecasting another. This way, and due to the inherent time lag in these models, this method is better suited to identify shock contagion links. [32] propose a slightly similar approach where they estimate shares of forecast error variances in time series resulting from shocks happening elsewhere.

While most studies examine correlation or causality between growth rates or levels of several macroeconomic variables like output or consumption [26–29], there are some examples where these time series are decomposed into a trend and a cycle component [25, 32]: the former captures long run tendencies while the latter reflects short run fluctuations or business cycles.

One of the main motivations behind these works on business cycle synchronization is to reveal the effect of globalization and international trade on shock contagion. Economic theory is not unambiguous about this question as trade itself may lead to more contagion and synchronization but specialization arising from trade may act in the other direction [26]. The empirical evidence in this respect is mixed, with evidences about a positive correlation between trade and increased synchronization of business cycles remaining at most limited [26, 27].

Although these works implicitly take into account pairwise correlation or causality among the economic activity of different economies, they do not refer explicitly to the topological aspects of the network which is built up by these pairwise relationships. [31] draw a map of causality links between five Asian economies, but the links identify association between sovereign debt risk of these countries and not standard macroeconomic variables. When estimating the relationship between industrial production of six of the G-7 countries, [32] report pairwise effects, but their analysis is aggregated and focuses on the overall connectedness (density) of this network which is broken down to the country level at most. More sophisticated network scientific tools were applied to business cycle synchronization by [33, 34], while these studies still focus on the density of the network with different techniques.

In this paper we fill this gap and provide a topological analysis of the global macroeconomic shock contagion network in an attempt to have a different perspective on the issue compared to previous studies. Topology may reveal some aspects of shock contagion which pure synchronization studies did not capture. In this attempt we use Granger causality tests as in [29] and [31] to identify shock contagion between countries. However, we use a wider set of countries than [29] and we use output data in contrast to risk measured by debt spreads in [31]. Contrary to most of the studies which use growth rates or level variables, we follow the logic of [25] and focus our analysis on the cyclic component of economic output, thus capturing causality effects between business cycle dynamics. Applying a rolling time window approach as in [32] we can give an overview about the evolution of this contagion network from 1961 to 2019.

The topological analysis is driven by the question whether shock contagion has a random pattern or not. Along this line, we relate the observed topological properties of the contagion network to those emerging from random networks. This way we can test whether contagion links are arranged systematically among countries or they appear randomly. Due to the longitudinal nature of our approach, a similar analysis can be applied on the level of pairwise connections with respect to the stability and systematic nature of shock contagion between pairs of countries.

In the next section of the paper we shortly describe the data preparation process and the methodology of network construction as well as the background of the topological analysis. Then, we present the main results of our analysis focusing on the evolution of topological properties first and the stability and systematic nature of pairwise connections second. As a third issue, following [26–28, 35] we give a simple outlook on the relationship between connectedness in the contagion network and economic openness.

## Materials and methods

### Data and preparation

This study uses GDP growth rates as a starting point. Data on the percentage change in the volume of economic output is available for a relatively wide set of countries and for a long period of time. In order to have a fine-grained picture on the economies under question and to have as many data points as possible, we use quarterly data as common in macroeconomic analysis. Data collected by the OECD is used covering OECD countries except Turkey, plus Bulgaria and Romania. Details of the data sources and preparation process can be found in S1 Table.

Data preparation starts with real GDP growth rates *r*_*t*,*i*_ for every quarter *t* and country *i*. These growth rates are then chain multiplied to get GDP-levels: $g_{t,i}=\prod_{\tau =1}^{t}\left(1+r_{\tau ,i}\right)$. In line with standard macroeconomic analysis, we use Hodrick-Prescott (HP) filtering in order to decompose individual time series of economic output into a trend (*y*_*t*,*i*_) and a cyclic (*c*_*t*,*i*_) component: *g*_*t*,*i*_ = *y*_*t*,*i*_ + *c*_*t*,*i*_. While the former corresponds to a long run steady path of the economies (also known as potential output), the latter shows fluctuations around this path. Our method builds on the cyclic components *c*_*t*,*i*_ which shows the extent to which country *i* is above/below its potential output in period *t* (the output gap). In order to control for differences in the magnitude of GDP levels and that of the resulting cyclic component, we use the percentage deviation between the cyclic and trend components in further calculations: ${\hat{c}}_{t,i}=c_{t,i}/y_{t,1}-1$.

Although there has been criticism about using the HP-filter in detrending macroeconomic time series [36], other studies still argue that the HP-filter can be applied meaningfully [37, 38] while [39] even shows that the HP-filter is appropriate for time series with more complex dynamics, especially in the case of GDP, which is used in the present paper. Moreover, while concerns about the HP-filter mostly targets its ability of real-time forecasting, we use the method in a retrospective manner which is more in line with its capabilities [40].

As a result of this data preparation process, we end up with time series of the relative cyclic components ${\hat{c}}_{t,i}$ for every country *i* in our sample. These series reflect the position of countries in their business cycles showing short-run fluctuations around their own trends. As these long run trends basically come from endogenous processes of the economies like resource accumulation and technological progress, focusing on the short run cyclic processes restrict our attention to those factors which are relevant from the perspective of shock contagion.

### The network

Moving beyond standard correlation studies, we use causality tests to construct the global contagion network as in [25, 29]. We run Granger causality tests for all pairs of countries using the cyclical components ${\hat{c}}_{t,i}$ as input time series. Granger causality tests allow us to see if one of the two time series helps to predict the other one. More formally, testing for Granger causality in our setup means estimating the following two regression models for every pair of countries *i* and *j*:

$$\begin{array}{c} {\hat{c}}_{t,i}=\beta_{0}^{1}+\sum_{l=1}^{L}\beta_{l}^{1}{\hat{c}}_{t-l,i}+\varepsilon_{t,i} \end{array}$$ (1)

$$\begin{array}{c} {\hat{c}}_{t,i}=\beta_{0}^{2}+\sum_{l=1}^{L}\beta_{l}^{2}{\hat{c}}_{t-l,i}+\sum_{l=1}^{L}\gamma_{l}{\hat{c}}_{t-l,j}+\mu_{t,i} \end{array}$$ (2)

While Eq (1) estimates an autoregressive process for country *i* with lags *L*, Eq (2) allows to test whether adding the past observation on country *j*’s cyclical position helps to better predict the cyclic position of country *i*. Having estimated these models, we calculate the F-statistic for all country-pairs as

$$\begin{array}{c} F_{i,j}=\left(RSS_{1}-RSS_{2}\right)/RSS_{2}\left(N-L\right)/L \end{array}$$ (3)

where *RSS*_1_ and *RSS*_2_ are the residual sum of squares of models Eqs (1) and (2) respectively, while *N* is the sample size. Given these test statistics we can calculate the probability of these F-values (*P*(*F*_*i*,*j*_)) to test whether the lagged cyclic position of country *j* significantly contributes to explaining the variance of the cyclic position of country *i*. In other terms, a significant F-statistic shows that if for some reason country *j* deviates from its long-run equilibrium path (the trend), this affects the economic circumstances (the cyclical position) in country *i*.

Causality, as defined above, provides the basis for our contagion network. Given that causality is revealed between country *j* and *i*, we define a link pointing from country *j* to country *i* in the contagion network. While mapping a network on this basis, we need to run lots of hypothesis tests on partially overlapping samples. This raises the problem of overestimating the number of network connections due to pure chance, through the accumulation of falsely rejected null-hypotheses (type I error). In order to control for this, we used the FDR method proposed by [41] and implemented the code developed by [42]. The adjacency matrix **A** of the contagion network is formally defined as

$$\begin{array}{c} a_{i,j}=\{\begin{array}{cc} 1, & \text{for}\;P\left(F_{i,j}\right)<f_{i,j},i\neq j\\ 0, & \text{otherwise} \end{array} \end{array}$$ (4)

where *f*_*i*,*j*_ is the test-specific critical value of rejecting the null-hypothesis. We used a 5% FDR in the analysis which means that we accept 5% of the rejected null hypotheses be false so that the null is true.

Two notes must be made here. In order that Granger causality tests yield accurate results, the time series used in the regression models must be stationary. Although the decomposition into trend and cycle mostly ensures stationarity in the cyclic component by the logic of the method, we explicitly tested stationarity of these time series. Stationarity is accepted for all time series in our sample—detailed results of these tests can be found in S1 Table.

The second note refers to the lags *L* used in models Eqs (1) and (2). We employed the Matlab code of [43] to run the causality tests which uses the Bayesian Information Criterion to determine optimal lag length. These optimal lags were used in the tests and can be found in S2 File.

We used Matlab to run the estimation procedure. The codes which handle the data and call the estimation procedure can be found in S1 File.

### Time handling

When it comes to data coverage of macroeconomic time series, one needs to face a trade-off between the spread (geographic coverage) and the length (time coverage) of the data: a wider set of countries can be analyzed if the time span is shorter, but a long time span needs restrictions on the set of countries included due to data availability issues. We address this trade off by using two distinct data sets. One of the data sets, referred to as the *Long dataset* starts with the second quarter of 1961 ending with the third quarter of 2019 and covers 25 countries. The second data set, referred to as the *Short dataset* includes 42 countries and covers the period between the third quarter of 1996 and the third quarter of 2019. The list of the countries included in the two data sets can be found in S1 Table.

The method described in the previous section can be used to construct the contagion network on time series of any length given that they are long enough to provide reasonable estimates. The length of our data sets (234 and 93 quarters) allows for the separation of time series into several subsets. In order to use as much information as possible, we apply the rolling time window approach as e.g. in [32]. This means that the estimations in models Eqs (1) and (2) are run on consecutive subsets of 52 quarters (13 years) in our sample. This allows for reliable estimates from the aspect of the number of data points involved, while also helps limiting the estimation to relatively homogeneous periods with as few structural breaks as possible (see e.g. [27]). This rolling time window approach provides basis for a longitudinal analysis of the contagion network.

From a formal perspective, the models in Eqs (1) and (2) are estimated for periods [*τ*, *τ* + 51] where *τ* goes from 1961Q2 to 2006Q4 in the *Long dataset*, and from 1996Q3 to 2006Q4 in the *Short dataset*. As a result, the adjacency matrices defined in Eq (4) refer to given time windows labeled with *τ*. **A**_*τ*_ thus reflects the contagion network observed in time window *τ*. The first time window covers the period between 1961Q2 and 1974Q1, the second the period between 1961Q3 and 1974Q2 and so on. The *Long dataset* covers 183 time windows while the *Short dataset* covers 42 time windows. As a result, we are able to analyze the dynamics of these snapshots of the contagion network over time.

### Measuring topology and dynamics

The basis for our analysis are the **A**_*τ*_ adjacency matrices which describe the shock contagion networks for a given time window *τ*. These networks are binary, directed and possibly change over time. This study focuses on the dynamics of these networks. In order to have a comprehensive picture, we first study the topological evolution of the network over time and then focus on the dynamics of pairwise connections. The applied metrics are introduced shortly below.

#### Network topology

In order to describe the (evolving) topology of the contagion network, we analyze the following properties of the network. More details of these methods are available in S1 Appendix.

*Density*. By density we mean the observed number of connections in the network, relative to the maximum possible number of links. Formally, the density for a given time window *τ* is calculated as *d*_*τ*_ = ∑_*i*,*j*_ *a*_*i*,*j*,*τ*_/(*N*^2^ − *N*). In our context density reflects how frequently shocks spread from one country to another. A more dense network would reflect frequent shock-transmission between countries: high exposure to shock originating in other economies as well as high probability of transmitting local turbulences overseas. The *igraph* package by [44] in R was used to calculate densities.

*Transitivity*. Transitivity captures the extent to which connections tend to cluster in the network. Formally, local transitivity or clustering of a node is defined as the share of closed triangles within all the triplets given by the node and its direct neighbors. Averaging over nodes we get global transitivity. In our context transitivity reflects the likelihood with which closed loops of shock contagion appear around countries so that if country A affects country B and country B affects country C then it is likely that country C also affects country A. High transitivity of this kind means that shocks originating in a country can reinforce itself by short ‘transitions’ through other countries. Given that our contagion network is directed, we use the solution of [45] and the R package *DirectedClustering* to calculate clustering.

*Path length*. Average path length captures how far nodes typically are from each other in the network. We calculate pairwise path lengths using the *igraph* package by [44] in R. Isolated pairs are assumed to be separated by a distance of *N* + 1. We normalized the direct path length counts to the [0, 1] interval. If *p*_*i*,*j*,*τ*_ is the shortest path length between *i* and *j* in time window *τ*, then the normalized average path lengths are obtained as ${\bar{p}}_{\tau }=\left(\sum_{i,j}p_{i,j,\tau }\right)/\left(N^{3}-N\right)-1/N$. Average path length in our context reflects how quickly shocks can spread in the global economy.

*Skewness*. In line with standard topological analyses, we are interested in the scale-free properties of the network. This is typically examined by fitting a power-law on the tail of the degree distribution [4]. As the size of the network in our case is rather small (25 or 42 nodes), standard techniques designed to estimate power law exponents can not be convincingly applied as these methods require a magnitude larger size at least. Therefore, we concentrate on the skewness of the degree distribution which is able to detect asymmetry. Significant negative (left) skew indicates that the majority of the nodes have relatively many connections why the minority has few. Significant positive (right) skew on the other hand shows few nodes with high degree and the majority with low degree—this is the case when the network exhibits similar structural properties as scale-free ones.

*Modularity*. Modularity captures the extent to which a network falls apart into sparsely connected groups or communities with relatively higher inside densities. In this study, we use the cluster-louvain method in the *igraph* package by [44] in R to partition networks into modules, which maximizes modularity to provide an appropriate partition. This required the transformation of the directed connections into undirected ones, which was achieved by using an edge weight of 2 in the case of bidirectional connections, 1 in the case of one-directional connections and 0 otherwise.

#### Network dynamics

Topological properties are able to describe a snapshot of a given network. In order to gain more insight into network dynamics, we also measure and analyze how pairwise connections evolve over time. We use two approaches along this question, which are briefly introduced below. More details are found in S1 Appendix.

*Transition probabilities*. Given the binary nature of the contagion network, there are four possible cases of transition: a previously non-existing link forms, an existing link dissolves, an existing link remains in the network and no link forms in the place of a non-existing link. For all time-windows in our sample we measure the share of the four transition cases. This allows the estimation of how stable connections are in our sample, shading the results from simple density as the latter being constant may arise from stable connections but also from the same amount of connections, but rewired.

*Systematically existing connections*. Transition probabilities can describe the overall dynamics of link formation in the network, but we can also exploit the dynamic information on pairwise connections. The connection between every pair of countries is described by a sequence of zeros and ones over time (collected in the adjacency matrices **A**_*i*,*j*,*τ*_). Applying the Wald-Wolfowitz runs test on this sequence we can test if the occurrence of shock contagion between two countries is purely random or systematic. With this method, we compress the dynamic information in our data and end up with an adjacency matrix where connection between two countries is defined if shock contagion is observed significantly more often than random *and* systematically spanning longer periods of time (compared to random occurrences).

#### The ER random graph as reference

In line with the main goal of this study, we test whether the different topological and dynamic characteristics of the observed contagion network differ from what is expected in a random network—i.e. if there are some systematic, predictable features in these networks. To operationalize this idea, we use the standard Erdős-Rényi (ER) random graph model as a reference point [46]. For all statistics calculated, we simulate 1000 independent directed ER graphs with the same size and expected density as in the observed network and then depict the observed statistics against the average values and 5%-95% percentiles calculated from the simulated random networks. This way we can test whether the given characteristic of the observed network differs significantly from what arises in a random network.

Another standard approach is to use the configuration model (CM) as a reference network, proposed e.g. by [4], which preserves the degree distribution of the observed network. In our context, though, this model has some limitations. First, we examine whether the appearance of shock contagion links between countries are random or they show some regularities or structure. The ER model replicates a ‘random world’ where this structure is not present, so any deviation from it is important in our context, even if it is the degree distribution which is shaded by the comparison with the CM. Second, correcting the CM for self-loops and multiple edges results either in a bias in network density when deleting the latter type of links or tremendous demand for computational power if one wants to keep only the feasible networks. However, in order to augment our results with the ER reference network, we also present the results with CM networks as the reference in S1 Fig.

## Results and discussion

### The evolution of topological properties

**Fig 1:**
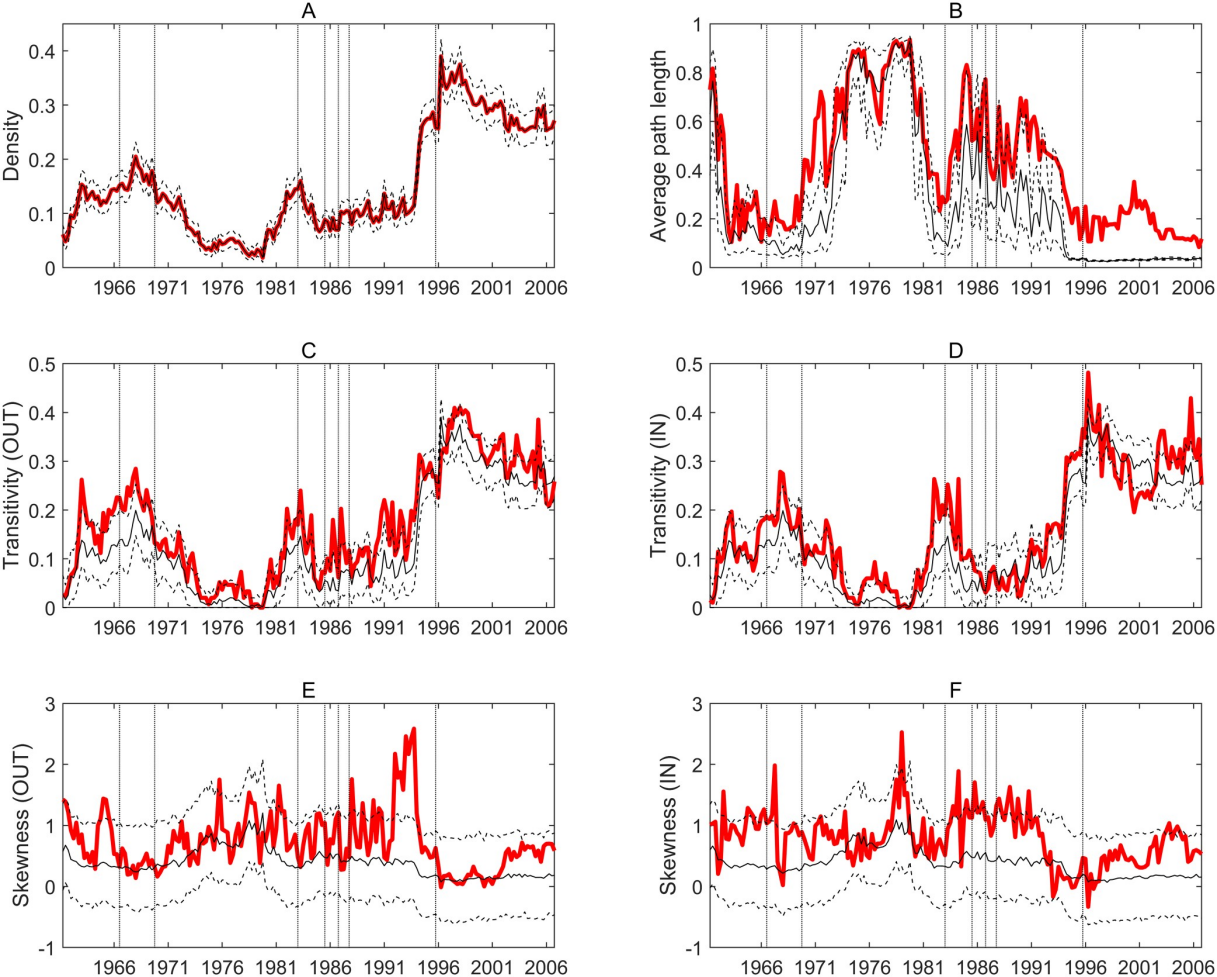
Topological characteristics of the global economic contagion network. Every panel shows the evolution of a topological property. Red lines mark the observed values, solid black lines mark the average from simulated ER networks while dashed lines represent the 5 and 95 percentiles of the latter. Horizontal axes show time windows with the labels marking the initial year of the given window (e.g. the last label refers to 2006 which means that this data point represent the topological property of the contagion network estimated on the 52 quarters starting from 2006Q1. Vertical dotted lines mark those time windows in which a given crises first appears. A: Density. B: Average path length. C: Transitivity (transmission). D: Transitivity (exposure). E: Skewness (transmission). F: Skewness (exposure).

The following analysis focuses on different topological properties of the global shock contagion network, using the *Long dataset* as it allows for a historical overview on its evolution. Fig 1 shows the main results with the red lines marking the observed topological characteristics and the thin black lines showing the average together with the 5 and 95 percentiles from the respective ER networks. These reference lines allow to test whether certain properties differ significantly from what is expected in a random network/world, where shock contagion happens by chance.

The evolution of density (panel A in Fig 1) shows a relatively sparse network. Density moves between 10 and 20% most of the time but it increases above 30% in the end of the sample period. It is interesting to see that in periods when crises hit the global economy (the vertical dotted lines) density tends to be higher than otherwise. However, the latest financial crisis in 2009 (the first occurrence of which in the estimated contagion networks is marked by the last vertical line) seems to have a more pronounced effect on density: it increases significantly and with a slight decline it remains at this higher level until the end of our sample. Also, a considerable increase in network density can be observed even before the crisis periods start to influence the estimations.

Although comparison with the ER network is irrelevant in the case of density (as the reference networks are constructed given the density of the observed networks), the remaining topological properties can be meaningfully compared to the random network. Panel B in Fig 1 shows that normalized average path length is quite volatile over our sample, driven by the density of the network: when density decreases, average path length increases and vice versa. This is explained by several isolated country-pairs which occur more often under lower density. On the other hand, average path length mostly remains within the range which characterizes random networks. Systematically positive deviations are found when network density is higher: in these periods the observed contagion network shows significantly more isolated country-pairs than the random network, which marks that shocks spread less rapidly in the observed network than it would be expected by pure chance. This pattern is the most visible during/after the 2009 financial crisis where density is also the highest. These conclusions are also backed by the phase-transition threshold of the ER graph. This threshold is around 4% density in our observed network with 25 nodes. When density drops below this threshold, the observed and reference networks falls apart into isolated groups which naturally increase path lengths. However, when density grows above this threshold, isolated components/nodes still remain in the observed network while disappearing from the random ones. As a result, significantly above-random path lengths are observed in these periods.

Panels C and D of Fig 1 show that transitivity does not differ significantly from random networks, except in a few periods. Transitivity in out-degrees is above-random in the end of the sixties while transitivity in in-degrees is above-random in the mid eighties. These periods overlap with those where longer average path length can be observed. However, the higher-than-random path lengths in the end of the sample are not matched with systematically higher-than-random transitivity. This points to some evidence that in those periods when observed network density is relatively high, path lengths and transitivity are likely to exceed that of a random network. This means that in these periods shocks tend to spread within local groups of countries, while transmission across these groups is less frequent, contributing to longer path lengths on average. There is no important difference between transitivities calculated on the basis of outgoing versus incoming links.

Similar to transitivities, we do not observe systematic tendencies with respect to skewness apart from a few periods (panels E and F of Fig 1). This means that there is no important asymmetry in the degrees so we do not detect either scale-free properties or the opposite, when having many links is general across nodes.

Fig 1A in S1 Fig shows similar tendencies when the reference network is generated from the configuration model, preserving observed degree distribution. As path lengths remain in the random range in this case and there is no skewness in the degree distribution, the higher-than-random path lengths can indeed be attributed to more isolates in the observed shock contagion network than expected randomly. On the other hand, transitivities follow similar patterns with the CM graphs as with the ER graphs which shows that this pattern is not a result of the specific degree-distribution of the observed network.

To sum up, these observations on the evolution of network topology show that

- density tends to be higher in crisis periods with the latest financial crisis in 2009 having a huge impact on density;
- shock contagion is quite close to random from a topological point of view. However, in periods with relatively higher density shocks tend to spread within local groups more than random (transitivity) but transmission across groups is less rapid (path length);
- there is no sign of asymmetric degree distribution (scale-free properties).

#### Robustness for crisis periods

As shown previously, density tends to be higher in crises periods. This calls for caution in handling our results so far. Given our methodology which basically checks lagged co-movement between business cycles of countries, we may be aware of the fact that a crisis hitting many countries can contemporarily push these cycles to the same direction, resulting in correlated cycles which then shows up as rejecting the no-causality hypothesis. In other words, periods of crisis may count as special outliers in the sample which may cause observed association. In such circumstances it is important to control for these outliers and check whether observed association (shock contagion) is kept if outliers are excluded from the estimation. Even more, the HP-filter which is used to construct the business cycles naturally creates ‘swings’ around crisis periods: positive output gaps before the crisis and negative ones during and after. In the case of a global crisis this leads to co-movements in all filtered time-series, increasing observed network density, as output gaps synchronize. In sum, excluding crisis periods from the estimation drives attention on the long run tendencies of the shock contagion network by eliminating the effect of common responses to global shocks around crisis periods.

In line with this argument, we run a robustness check by recalculating the adjacency matrices of the shock contagion networks excluding the crisis periods from the estimation of models Eqs (1) and (2). The omitted crisis periods can be found in S1 File and the method of these estimations is described in S1 Appendix.

**Fig 2:**
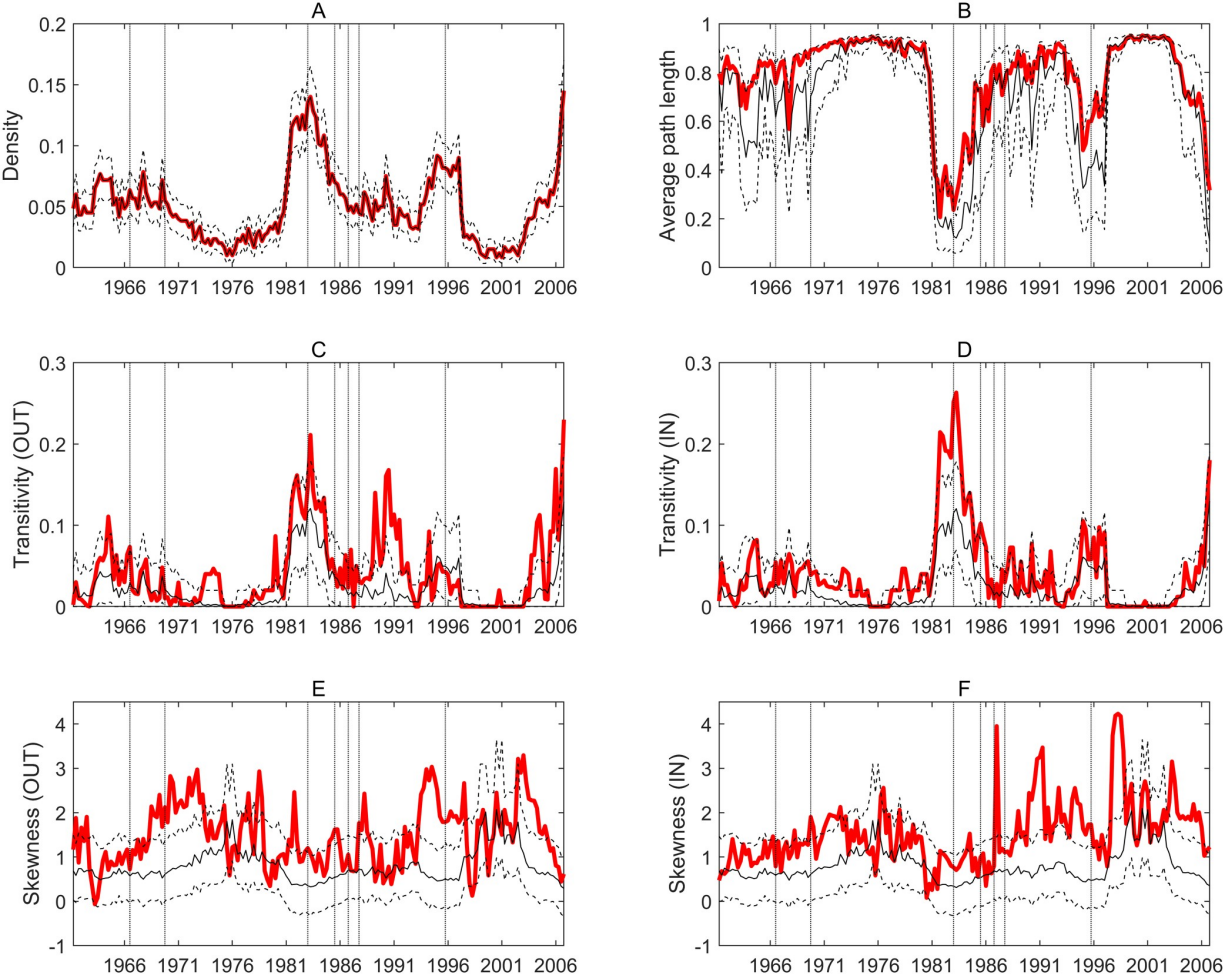
Topological characteristics of the global economic contagion network with crisis periods excluded. Every panel shows the evolution of a topological property. Red lines mark the observed values, solid black lines mark the average from simulated ER networks while dashed lines represent the 5 and 95 percentiles of the latter. Horizontal axes show time windows with the labels marking the initial year of the given window (e.g. the last label refers to 2006 which means that this data point represent the topological property of the contagion network estimated on the 52 quarters starting from 2006Q1. Vertical dotted lines mark those time windows in which a given crises first appears. A: Density. B: Average path length. C: Transitivity (transmission). D: Transitivity (exposure). E: Skewness (transmission). F: Skewness (exposure).

Fig 2 shows the same topological properties as analyzed before, now calculated from the adjacency matrices estimated without the crisis periods. The structure of Fig 2 is the same as that of Fig 1. For easy comparison, we also left the vertical lines in Fig 2.

The most important observation from Fig 2 is that up to the last vertical line, marking the financial crisis in 2009, the patterns are very similar. This is due to the fact that all the previous crises were shorter (typically two or at most four quarters are excluded) and affected seriously only a subset of the countries. On the contrary, the 2009 financial crisis hit all countries in the sample and typically to a large extent while being longer, thus resulting in the elimination of 10 subsequent quarters from the estimations. These all explain the significant difference between Figs 1 and 2 for the time windows containing this crisis.

It is important to note that the density of the crisis-controlled contagion network is significantly smaller (around 5-10%) than that of the uncontrolled network (panel A of Fig 2). On the other hand, the patterns are quite similar with a massive valley in the seventies and then a sharp increase in the beginning of the eighties. Also, apart from the few quarters around the financial crisis in 2009, we can reveal a steadily decreasing trend in the density from roughly the mid eighties. This means that ruling out the naturally increasing correlation between business cycles in crisis periods, the contagion network became sparser during the end of the 20th century. However, in the last periods of the sample density rises again, which is in line with the observation in Fig 1 where the 2009 financial crisis heavily increased density, but then remained at a high level. These results with the crisis periods excluded shows that this high level of density at the end of the sample was not exclusively a result of natural co-movement between business cycles in a global crisis, but also arises from long-term cyclical patterns of business cycle synchronization.

This result further refines previous studies which found no robust evidence on globalization bringing more synchronized business cycles in the past decades. Our observations show that while this synchronization decreased during the last decades of the 20th century and the first decade of the 21st, it started to rise again in the most recent decade. This, together with our other results, shows that global synchronization follows its own cycles irrespective of temporary shocks. Still, the 2009 financial crisis had a significant impact on the density of the contagion network, although temporarily.

When looking at average path length (panel B of Fig 2) and transitivities (panels C and D of Fig 2) the main conclusion remains the same as without controlling for crisis periods: both path lengths and transitivity are significantly higher in the observed networks than in a random one in some, typically overlapping, periods. On the other hand, there are very few such periods as the density of the crisis-controlled network falls below the threshold of connectedness around 4% quite often.

In the case of skewness, we see a significant positive asymmetry in the end of the sample (panel E and F of Fig 2). This means that the degree distribution contains more nodes with relatively less connections while there are not as many nodes with relatively many connections. This is true for both the indegrees and the outdegrees (but it is more pronounced for the latter), so we observe a scale-free-like structure in the contagion network roughly in the past 20 years: there are few countries which are relatively more exposed to or transmitters of shocks, while the majority has relatively lower exposure and ability to transmit shocks. These results are reinforced by Fig 2A in S1 Fig, with the CM graphs used as reference.

To sum up, controlling for crisis periods (excluding them from the estimation procedure) have shaded our results by showing

- decreasing tendency for network density, thus overall shock propagation frequency from the mid 80s until the beginning of the 21st century;
- the latest financial crisis in 2009 still have significant, but temporary effect on network density;
- although the network becomes quite sparse with many isolated groups, once crisis periods are excluded, periods with higher density still exhibit relatively high average path lengths and transitivity, marking the presence of local groups;
- degree distribution is asymmetric in the 90s and early 2000s, which resemble scale free properties.

### Link formation dynamics

In the previous section we analyzed how topological properties changed over time within snapshots of the contagion network. Now, we focus on link dynamics as it was described previously. First, Table 1 shows average transition frequencies between the two possible states of connections (existing and non-existing), for the *Long dataset*, by depicting the frequency of different transitions in the sample. Frequencies are calculated with and without the crisis periods in the sample and the results are contrasted to the frequencies obtained from random networks.

**Table 1:** Average frequency of transitions in the *Long dataset*, compared to random networks.

Crisis periods included			Crisis periods excluded	
	*Existing*	*Non-existing*	*Existing*	*Non-existing*
*Existing*	13% / 3%	2% / 11.5%	4% / 0.5%	1% / 4.5%
*Non-existing*	2% / 11.5%	83% / 74%	1% / 4.5%	94% / 90.5%

Percentages reflect the observed share of different state transitions / the same share in random networks with the same density.

The most important result in this respect is that the observed contagion networks are more stable than expected in a random network: shock contagion links between countries tend to dissolve less frequently which shows some systematic relationship between these pairs of countries. We may also evaluate stability from the aspect of transition probabilities, by row-standardizing cells in Table 1. Results in Table 2 show these transition probabilities in the same manner as the frequencies in Table 1. The numbers there reflect the probability with which connections end up in one of the (column) states *given* the previous (row) state. These results reinforce that connections are more stable than in a random network, so connections are persistent in the sense that given they are formed, they dissolve less frequently than expected in a random network.

**Table 2:** Average transition probabilities in the *Long dataset*, compared to random networks.

Crisis periods included			Crisis periods excluded	
	*Existing*	*Non-existing*	*Existing*	*Non-existing*
*Existing*	85% / 15%	15% / 85%	80% / 5%	20% / 95%
*Non-existing*	2% / 15%	98% / 85%	1% / 5%	99% / 95%

Percentages reflect the observed share of different (column) transitions from a given (row) state / the same share in random networks with the same density.

**Fig 3:**
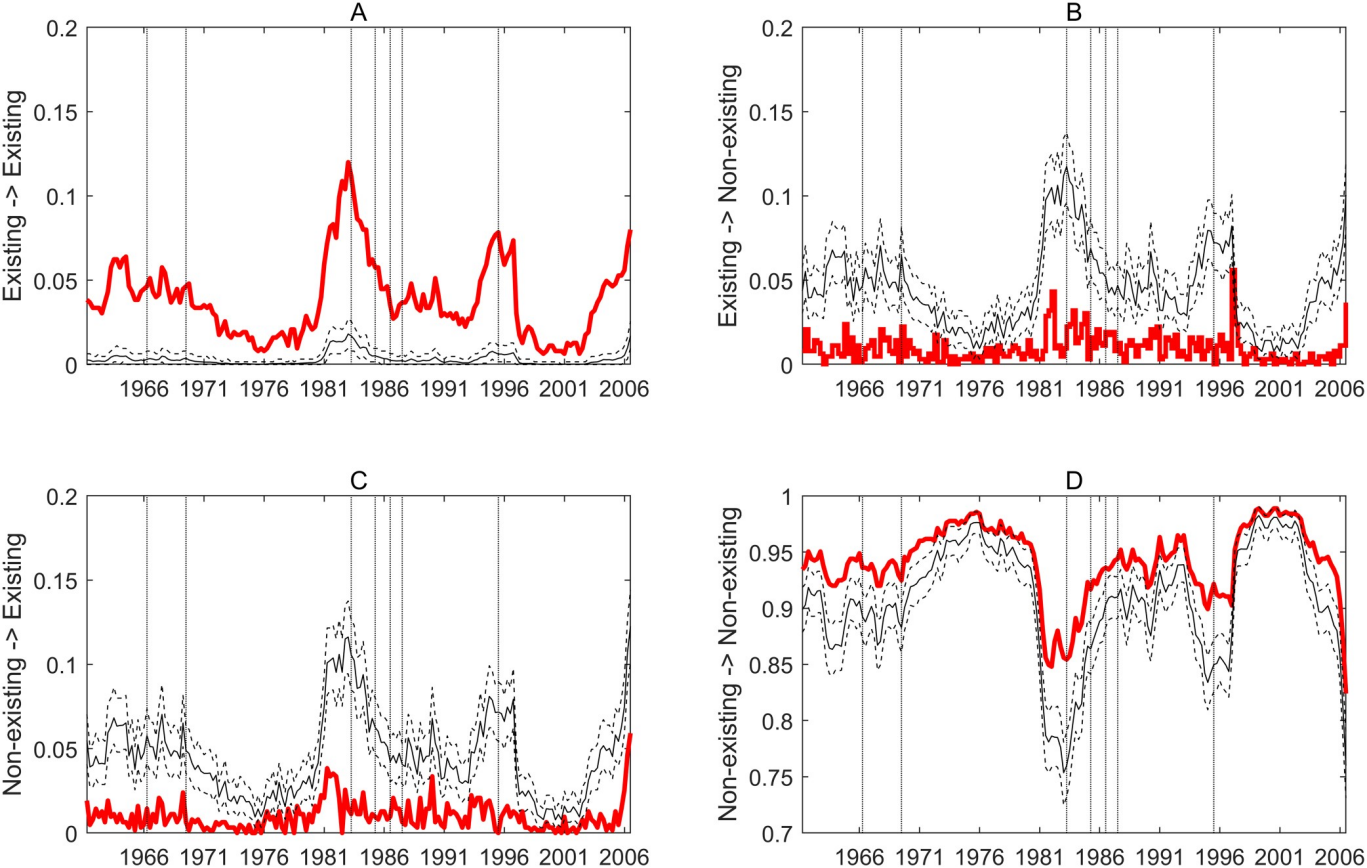
Link formation dynamics in the global economic contagion network. Every panel shows the path of a state transition frequency, where states refer to whether a connection exists or not. Red lines mark the observed values, solid black lines mark the average from simulated ER networks while dashed lines represent the 5 and 95 percentiles of the latter. Horizontal axes show time windows with the labels marking the initial year of the given window (e.g. the last label refers to 2006 which means that this data point represent the topological property of the contagion network estimated on the 52 quarters starting from 2006Q1. Vertical dotted lines mark those time windows in which a given crises first appears. A: Frequency of stable existing connections. B: Frequency of dissolving connections. C: Frequency of newly formed connections. D: Frequency of stable non-existing connections.

The previous analysis focused on the overall dynamics of the network—but the data also allows to show how these transition frequencies evolved over time. Fig 3 extends over time the average frequencies shown in the right hand side of Table 1 (crisis period excluded). These paths are calculated with the crisis periods omitted from the estimations and Fig 3 uses the same logic as Figs 1 and 2 by marking the respective transition frequencies in a random network.

The results reinforce the numbers in Table 1: observed frequencies are significantly higher in the stable transitions than in a random network, while significantly lower in the unstable transitions. This is true over the whole sample period. Although the low density of the network results in a very low probability of finding stable links by pure chance, the result of significantly more stable existing connections mean that there are country-pairs within which contagion is systematically more likely than in others. In other words, shock contagion links in this network are relatively persistent. On the other hand, the stable existing transitions clearly follow the pattern of network density shown in Fig 2: decreasing tendency over the last two decades of the 20th century and in the first decade of the 21st. Then, the last one and half decade is marked by increasing density and still systematically stable connections. Naturally, the opposite tendency is followed by the non-existing stable connections reflecting the network gradually getting sparser and then denser at the end. Interestingly, though, the unstable (forming and dissolving) transition frequencies are low, but stable even during the latest decades, apart from tha last few years. This shows that in spite of the whole contagion network getting sparser, link formation and dissolution keep their frequency. This results in a relatively more vibrant network in the past few decades, except the last few years. This is shown by the decreasing random network frequencies: observed unstable frequencies are still significantly lower than the random reference but the difference is getting smaller.

In summary, link formation probabilities show

- more stable (persistent) connections than it would arise from a random network;
- but a more vibrant (less persistent) connections structure in the last two decades of the 20th century and the first decade of the 21st.

### Systematic contagion paths

The previous dynamic analysis allowed us to examine the frequency at which contagion connections form and dissolve between countries. However, that approach aggregated this dynamics over country-pairs while keeping the time dimension explicit. In what follows, we make pairwise connections explicit, while compressing the time dimension: using the runs test described previously, we are able to detect those country-pairs where shock contagion happens in a systematic (non-random) way.

**Fig 4:**
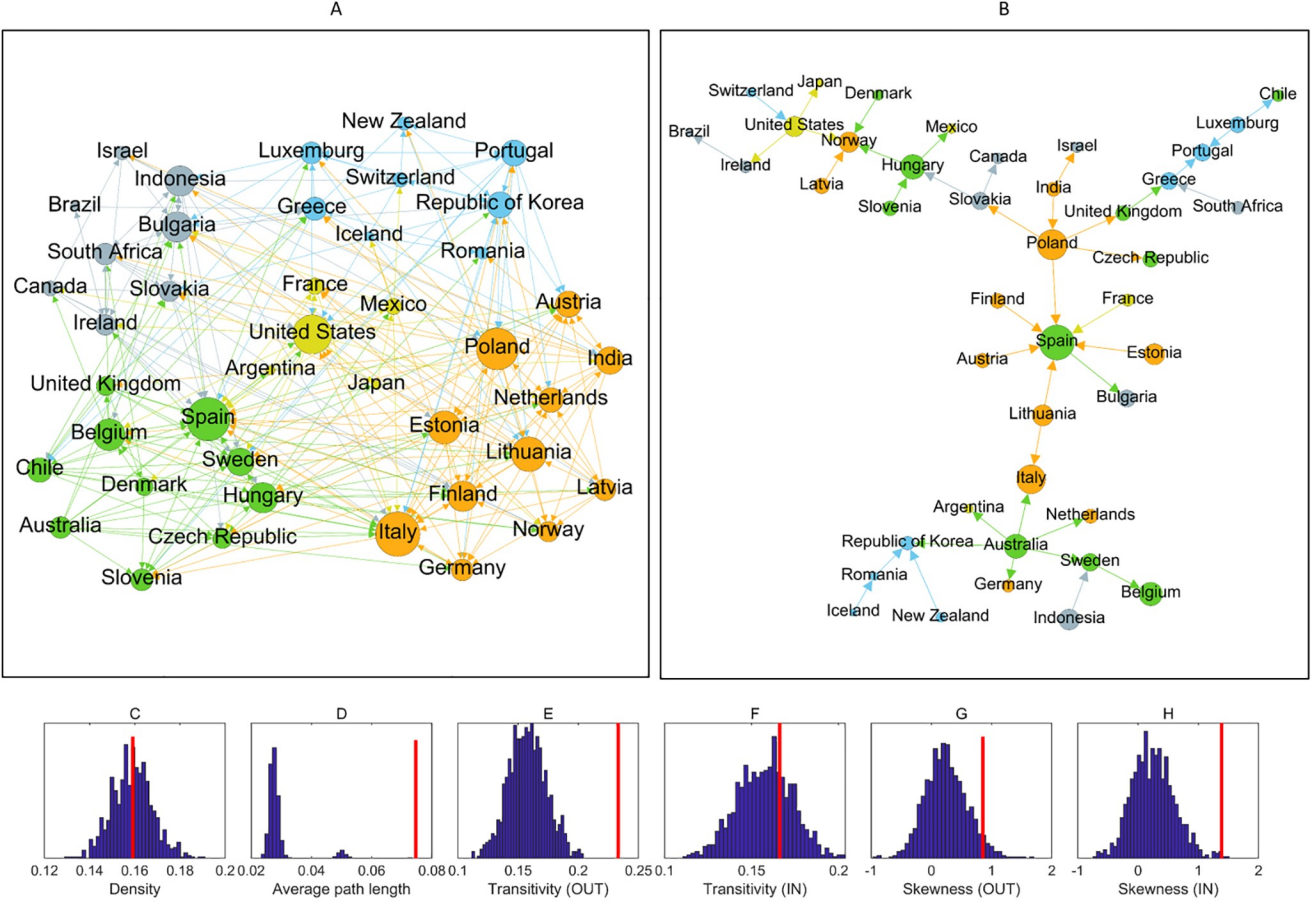
Systematic shock transmission paths in the global economic contagion network. Panel A shows the full network of systematically existing contagion paths. Node sizes reflect the sum of in- and outdegrees. Panel B shows the maximum spanning tree (MST) based on the systematically existing contagion paths. Coloring of the nodes in panels A and B reflect the modules. In panels C-H vertical red lines show observed topological properties in the mapped network while the blue histograms show the distribution of the same properties from a sample of 1000 independent ER random networks of the same density. C: Density. D: Average path length. E: Transitivity (transmission). F: Transitivity (exposure). G: Skewness (transmission). H: Skewness (exposure).

Panel A in Fig 4 shows the map of the contagion network obtained by this method. The links in this map refer to those directions in which shock contagion seems to happen systematically during our sample period. In this figure, we use the *Short dataset* in order to have a larger set of countries. Crisis periods are omitted from the estimations. Panel B shows the maximum spanning tree obtained from a weighted, undirected version of the same network. Details of the procedure behind this picture is given in S1 Appendix. The size of the nodes represent their degree, while coloring reflects the modules they belong to. Modules are identified by the method described earlier (details are also found in S1 Appendix). Panels C-H show the main topological properties of this network: vertical red lines mark observed values and the blue histograms show a sample of the same indicator resulting from ER random networks of the same density.

From the network map one can first see the relative density of the network. Being around 16%, this density (panel C) shows that on average countries are exposed to shocks from roughly one sixth of the sample countries. The topological gauges at the bottom of Fig 4 indicate that the average path length (panel D) and the transitivity in outdegrees (panel E) of this network are significantly larger than those of a random one. Contrary, transitivity in indegrees (panel F) seem not to differ from a random network. Skewness shows modest positive asymmetry, while it is more pronounced (tending towards being significantly different from random) in the indegrees (panels G and H). Transitivity is shown to be higher than random in outdegrees which means that there tend to be groups of countries within which shocks can spread relatively quickly, possibly reinforcing themselves. Skewness is also higher then random in both directions indicating the presence of asymmetry in the degree distribution: while relatively few nodes possess many connections, the majority is characterized by relatively less links. These topological properties are contrasted to the configuration model as reference network in Fig 4A in S1 Fig. The similar results in this case show that the topological properties observed are not arising from the specific degree distribution. Higher-than-random path length, out-transitivity and skewness all point to a modular structure where shock contagion is stronger within groups but slower accross groups, the latter contributing to relatively long path lengths. At the same time, the network is dominated by a few strongly connected nodes while the majority have less connections, resembling a scale-free structure.

Fig 4 also reflects how countries shape modules in the network. According to the community detection algorithm described earlier (details are also given in S1 Appendix), we can identify five distinguishable groups of countries marked by the different colors of the nodes. First of all, it is hard to identify an apparent organizing principle behind these groups: they are all heterogeneous both from a geographical perspective and from the viewpoint of the development level of their members. The orange group on the bottom-right part of the picture is geographically the most homogeneous, with European countries plus India. The yellow group in the middle is dominated by Americas, but there is France and Japan as well. The grey group in the upper-left part is the most global with countries from four continents.

Panel B with the maximum spanning tree highlights the most important links in this network. Although there is a tendency for the different groups to be close in this picture (linked by strong, systematic contagion paths), still they are fragmented in the sense that smaller sets of countries of the same module can be found relatively far apart in this network. On the other hand, it becomes visible here that the most central actors in this network are European countries, plus Australia and to some extent the US. The main organization of the maximum spanning tree shows a chain-like structure, which reinforces our previous observation about the relatively high path lengths in this network.

**Fig 5:**
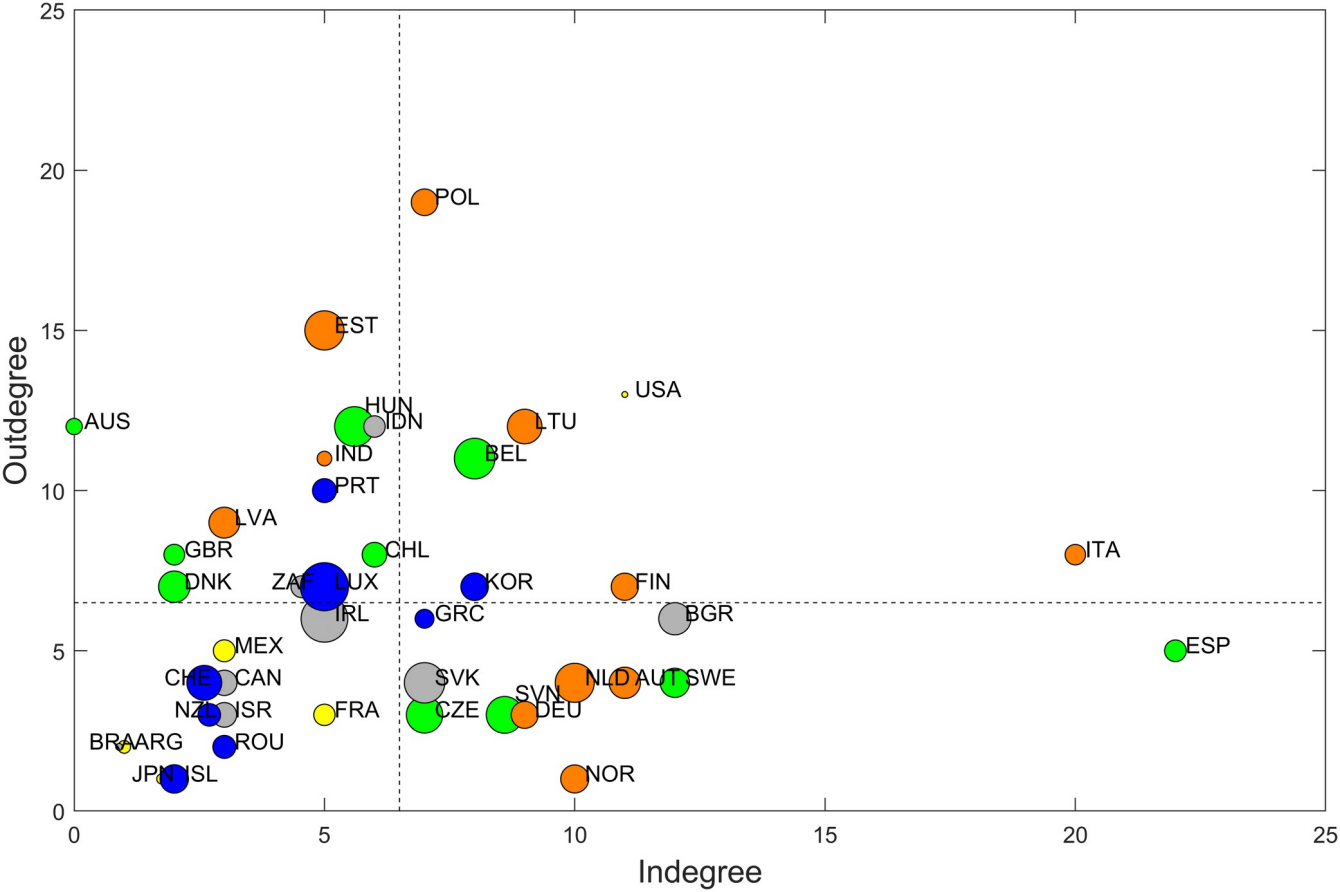
Trade openness and connectivity. Bubbles represent countries. Horizontal axis measures indegree in the contagion network depicted in panel A of Fig 4, vertical axis measures outdegree in the same network. The size of the bubbles are proportional to economic openness measured by exports plus imports as a share of GDP, averaged over the period between 1996 and 2019 to match the time span of the network. Coloring reflects the modules, the dashed lines mark average in- and outdegrees.

The structure of systematically existing contagion paths can be further analyzed with the help of Fig 5. Here, the countries are plotted according to their in- and outdegrees in the network shown in panel A of Fig 4. First, countries are roughly equally scattered in the four quadrants defined by average degree (marked by the dashed lines). 12 countries can be found in the lower-left quadrant where both in- and outdegrees are lower than average. Also 12 countries are in the upper-left quadrant: these countries have relatively low indegrees and high outdegrees, thus they are more likely to be transmitters of shock rather than recipients. 11 countries in the lower-right quadrant show the opposite tendency with high indegree and low outdegree. Finally, the least numerous group with 7 countries is the most central in the contagion network with both high in- and outdegrees. Looking at the composition of these groups we still not find strong organizing principles: all quadrants are heterogeneous with respect to geography and development level. However, the modules of the network show a bit more organized pattern: countries in the yellow, blue and gray modules show relatively balanced centrality: their in- and outdegrees are more-or-less similar. On the other hand, green and orange modules are placed dominantly in the upper-left and lower-right quadrants: these countries are characterized by an unbalanced centrality, being either transmitters or recipients of shocks in general.

Given that these first-cut analyses show few systematic patterns, understanding the principles behind the modular structure of this network requires further investigation.

In sum, analyzing systematically existing pairwise contagion links, we see that

- there are quite a few systematically existing contagion paths over time, with a country affecting or being affected by roughly 6.5 other countries on average;
- topological properties of this network show a modular structure and an asymmetric degree distribution;
- there is no apparent organizing principle behind the modules with respect to geography or development;
- part of the modules show balanced and others show unbalanced centrality in the directed network.

### Shock contagion and trade

Although this study concentrates on the topological properties of the shock contagion network, it is important to keep in mind that the observed/estimated shock contagion connections reflect deeper economic mechanisms which transmit processes and events from one country to another. A more profound analysis of these channels require further investigation, however, Fig 5 gives a first-cut overview of whether economic openness is related to the position of countries in the shock contagion network. Apart from the in- and outdegrees on the two axes and the modules reflected by coloring, the size of the bubbles reflect their economic openness as measured by the sum of export and import volumes relative to the Gross Domestic Product, averaged over the period between 1996 and 2019.

The first glimpse on Fig 5 does not reveal clear, monotonic tendency between openness and centrality in either direction. A closer look, however, shows that those countries which have the higher openness in our sample (Luxembourg, Ireland, Slovakia, Belgium, Estonia, Hungary) are found closer to the center of the figure, i.e. around the intersection of the two dashed lines. This means that these relatively open economies have a balanced position with respect to centrality: they show up as transmitters and also as receivers of shocks at a similar frequency. On the other hand, these countries do not show extreme centrality in either dimension, their connectedness is closer to average. In contrast, less open economies are more frequently found in the outer parts of the figure, which means that these countries are more likely to have relatively low or high connectedness in one of the directions or in both. This result indicates that more open economies are neither extremely exposed to nor are extreme transmitters of shocks. On the contrary: they have a relatively balanced position in the contagion network.

We have also checked whether the four quadrants on Fig 5 show significant differences in the openness of the countries which belong to each of the quadrants. We have calculated the average openness for the countries in all four groups. These average openness values are shown in the respective Table 3, while the overall average is show in the lower-left cell. These values show that there is no striking difference between the group-averages and the overall average. This is reinforced by two-sample t-tests (in parentheses) which show no significant differences between the group means and the whole sample mean. Although economic openness is measured with total exports and imports in Fig 5, no significant association between openness and network position is found even if export and import volumes are calculated within those countries which are part of our sample (see Fig 5A in S1 Fig).

**Table 3:** Average openness by centrality groups.

	*Low indegree*	*High indegree*	*Average*
*High outdegree*	48.59% (0.4784)	40.58% (-0.2911)	44.58%
*Low outdegree*	34.25% (-1.2394)	48.19% (0.8270)	41.22%
*Average*	41.42%	44.38%	42.90%

Percentages reflect the observed share of exports plus imports in GDP within the given group of countries. The four groups correspond to the four quadrants in Fig 5. Numbers in parentheses show two sample t-statistics when the given group mean is tested against the overall sample mean (lower left cell).

To sum up the observations with respect to openness and contagion, we see that

- although there is a tendency for more open economies to have a balanced and non-extreme position in the contagion network;
- there seems to be no clear evidence on the relationship between economic openness and exposure to shocks.

## Conclusion

In this study we constructed networks of economic shock contagion by estimating Granger causality of output time series among countries. Using rolling time windows for the estimations, we were able to conduct a longitudinal analysis on the topology of this contagion network. The results show that the density of this network typically moves between 10 and 20% which is relatively high: on average countries are exposed to shocks from one sixth of the economies. Periods of economic crises seem to increase this density, however the latest financial and economic crisis proves to be special by increasing the density up to 40% and having a long lasting effect compared to previous crisis periods. When we eliminate the effects of crisis periods from the estimation, a clear negative tendency is observed in network density over the last two decades of the 21th century and roughly the first decade of the 21st. Then, the latest decade is again marked by a significant increase in density, this synchronization of business cycles. This means that disregarding crisis periods, shock contagion follows show a cyclic pattern: it became overall less frequent in the global economy since the beginning of the 1980s, but increases again over the last decade. This conclusion further refines our understanding about the relationship between business cycle synchronization and globalization.

We also found that in periods when its density is not extremely low, the contagion network shows longer path lengths and transitivity than respective random networks. This means that shocks tend to spread in local neighborhoods while crossing the boundaries of these neighborhoods is less likely. In the latest three decades the degree distribution of the contagion network tend to be asymmetric with relatively more countries having few links and the minority having many connections.

By looking at pairwise connections in the contagion network we observed more stable links than expected in a random network, however, the connections seem to become more vibrant at the end of our sample. We also extracted those country-pairs which show systematically (non-randomly) occurring contagion links over time. Here we again observed asymmetric degree distribution with a few countries having relatively many links and the majority having less. While there is a tendency for economically more open countries to have a more balanced and less extreme centrality in this network, we did not find evidence of a strong relationship between economic openness and exposure to shock contagion in either direction.

To sum up, this study reveals some non-randomness in the global shock contagion network: asymmetric degree distribution being one of the most robust results but we also found stable and systematically existing connections/shock contagion paths. On the other hand, we find no clear evidence of economic openness correlating with degrees, moreover, in the past few decades (marked with increasing globalization) the density of the shock contagion network seems to steadily decrease.

This analysis may be further refined in several ways. From a methodological point of view, more sophisticated ways of extracting business cycles can add to the mapping of the contagion networks. Also, the possible bias arising from the limited sample size can be tracked by incerasing the range of countries or using bootstrap methods on the given sample. Further research also calls for a deeper analysis of pairwise shock contagion patterns both from a geographical and historical perspective. Also, the role of trade relationships (sectoral composition, incoming and outgoing trade) has to be analyzed better. Finally, the organizing principles behind the modules observed in the contagion network must be understand better.

## Supporting information

S1 Appendix Methodological description. This document contains additional details on the data sources, the data preparation process and the methodology.
(PDF)

S1 Table Data tables and auxiliary results. These data sheets contain the original data on GDP growth rates. They also present additional data processing steps and present auxiliary information about the data handling process. The content and short description can be found on the first data sheet.
(XLSX)

S1 File Codes for the estimation process. These files contain the Matlab codes used for the rolling window Granger causality estimation procedure. These codes call the Granger causality test of [43]. The package also contains the data structures used by these codes in .mat files.
(ZIP)

S2 File Lag structure of estimations. These data structures contain the optimal lag structures used in the estimations. The optimal lags are given in separate .mat files for the *Long dataset* (labeled with 25 in the filenames) and *Short dataset* (labeled with 42 in the file names), for the crises-included and the crises-excluded (labeled with NC in the file names) and for the explained (labeled as Y in the filenames) and explanatory (labeled as X in the file names) country in the Granger tests. All files contain an array of *N* times *N* times *T* with *T* being the number of time windows and one entry showing the optimal lag of the given time series for the given country pair.
(ZIP)

S1 Fig Additional figures. These figures replicate Figs 1, 2, 3 and 4 with the configuration model as the randomized reference network. Also, Fig 5 is replicated with openness values restricted to trade within the sample countries.
(PDF)
