# Supplementary material for: Do economic shocks spread randomly?: A topological study of the global contagion network
Source: PLoS One. 2020 Sep 4;15(9):e0238626. doi: 10.1371/journal.pone.0238626 (PMC7473515; doi:10.1371/journal.pone.0238626)
Supplement: S1 Appendix — This document contains additional details on the data sources, the data preparation process and the methodology. (PDF) [file pone.0238626.s001.pdf]

1. Data

We downloaded quarterly percentage change (to the previous period) GDP growth data from 1960 to 2019 from the OECD database.

Source: OECD (2020), Gross domestic product (GDP) (indicator). doi: 10.1787/dc2f7aec-en (Accessed on 05 February 2020); Source: OECD National Accounts Statistics: Quarterly National Accounts

Two datasets were created for different analytical purposes:

The *Long dataset* covers a longer time period but involves fewer countries.

Time period: from the second quarter of 1961 to the third quarter of 2019.
25 countries: Australia (AUS), Austria (AUT), Belgium(BEL), Canada(CAN), Switzerland(CHE), Germany (DEU), Denmark (DNK), Spain (ESP), Finland (FIN), France (FRA), United Kingdom (GBR), Greece (GRC), Ireland (IRL), Island (ISL), Italy (ITA), Japan (JPN), Korea (KOR), Luxemburg (LUX), Mexico (MEX), Netherlands (NLD), Norway (NOR), Portugal (PRT), Sweden (SWE), United States of America (USA), South Afrika (ZAF).

The *Short dataset* covers a shorter time period but involves more countries.

Time period: from the third quarter of 1996 to the third quarter of 2019.

42 countries: Argentina (ARG), Australia (AUS), Austria (AUT), Belgium(BEL), Bulgaria (BGR), Brazil (BRA), Canada (CAN), Switzerland(CHE), Chile (CHL),Czech Republic (CZE), Germany (DEU), Denmark (DNK), Spain (ESP), Estonia (EST), Finland (FIN), France (FRA), United Kingdom (GBR), Greece (GRC), Hungary (HUN), Indonesia (IDN), India (IND), Ireland (IRL), Island (ISL), Israel (ISR), Italy (ITA), Japan (JPN), Korea (KOR), Lithuania (LTU), Luxemburg (LUX), Latvia (LVA), Mexico (MEX), Netherlands (NLD), Norway (NOR), New Zealand (NZL), Poland (POL), Portugal (PRT), Romania (ROU), Slovakia (SVK), Slovenia (SVN), Sweden (SWE), United States of America (USA), South Afrika (ZAF).

The downloaded and sorted data can be found in the worksheets of S2 Table.

2. Cyclical components of the time series

From the downloaded GDP growth data, we calculated GDP level data. We normalized data to a common starting point (100), and with the GDP growth rate we calculated the indexed data (on the 25 countries data: 1961:Q1 = 100 and on the 42 countries data: 1996:Q2 = 100) . The calculated GDP index data can be found in S2 Table.

$GDP_t = GDP_{t-1} + (GDP_{t-1} * GDP\_growth_t)/100$

We used the Hodric-Prescott filter to remove the cyclical component of the time series. For the filtering we used the hpfilter function in Matlab with the default smoothing parameter of 1600.

```
[T,C] = hpfilter(GDP, 1600);
```

We calculated the percentage deviation of the cyclical component from the trend component.

```
Y = (C./T-1)*100;
```

In the last step, we deleted the first observation of cyclical component (that was generated in the first step) and we have got the final Y time series (see Y.mat, Y42.mat in S3 File). This is labelled as $\hat{c}_{t,i}$ in the paper.

3. Stationarity test

To apply the Granger causality test, first we need to check for the stationarity of the time series.

We applied Kwiatkowski-Phillips-Schmidt-Shin (KPSS) test and Augmented Dickey-Fuller Test (ADF) for the whole time series and for the subsamples on the longer and shorter database.

We used R software, aTSA package for the tests.

We applied the kpss.test function, and we saved the type 1 results (no drift, no trend) in the respective worksheets of S2 Table. There we also summarized the results of the subsample tests.

We applied the adf.test function, and we saved the type 1 (no drift, no trend) results with lag = 1 in the respective worksheets of S2 Table. And we also summarized the results of the subsample tests.

4. Adjacency matrices

We used Granger causality to build the contagion networks. In the case that country "A" Granger cause country "B", we have defined a directed link from country "A" to country "B". To calculate the Granger causality we used the granger_cause_1 function in Matlab.

Source: *Robert (2020): Granger_Cause_1 (https://www.mathworks.com/matlabcentral/fileexchange/59390-granger_cause_1), MATLAB Central File Exchange. Retrieved February 24, 2020.*

In order to control for multiple hypothesis testing which results from simultaneously testing 25*25 and 42*42 hypotheses of Granger causality, we employed the FDR method. Practically the fdr_bh function was used in Matlab with a 5% FDR. The method was applied separately for all time windows.

Source: David Groppe (2020): fdr_bh (https://www.mathworks.com/matlabcentral/fileexchange/27418-fdr_bh), MATLAB Central File Exchange. Retrieved July 10, 2020.

We used rolling time window analysis. The rolling window size is 52 quarters. The rolling_Granger.m in S3 File runs on the Y time series and chooses every 13 years (52 observations) periods. The script uses the networkGranger.m function in S3 File that chooses the country pairs. The granger_cause_1 function is running under them, it calculates the F values and p-values of the Granger causality test, and the results are saved for each country pair in a matrix, and the matrices build a 3D array, where the third dimensions are the time windows. The main output of the rolling_Granger script is the Granger adjacency array with 0 and 1 elements according to the significance of the p-values.

To exclude crisis periods, we used the Omitperiods vector (see in S2 Table) and we modified the networkGranger.m function to networkGrangerNC.m found in S3 File. When the function chooses the country pair, it will filter those elements of the Y columns where the value of the Omitperiods vector is 1. The Omitperiods vector is a column vector with 0 and 1 elements. The element is 1, when there is a crisis period, and the first period after the crisis period in order to avoid crisis period both on the left- and the right hand sides of the estimated equations.

5. Identifying and excluding crisis periods

We used two methods to identify times of crises. First we checked the literature (e.g.: First oil crisis, Crisis of 2008). Where we did not find an exact identification of the quarters of the crises (e.g. Second

oil crisis, Latin-American crisis) we checked the time series, and we identified the initial quarter of the crisis where GDP growth declined for most countries in the year of the crises.

With these methods, we identify the following crises periods:

1973Q4: First oil crisis

1979Q2: Second oil crisis

1982Q3: Latin-American crisis

1995Q4: Mexican peso crisis

1998Q2: East Asian crisis

1999Q3: Russian crisis, Brazilian crisis

2000Q3: Dotcom crisis

2008Q3 - 2009Q3: Crisis of 2008

Once these crisis quarters are determined, we re-run Granger-causality tests without these quarters in the sample. In order to filter out crisis periods, we employ the fact that the regressions in equations (1) and (2) of the manuscript are cross-sectional in nature: the time structure of the data is imposed by 'shifting' the same data series on the right hand side compared to the left hand side. The data structure with lag 2 looks like as in the following table:

# of obs.	y_t	x_{t-1}	x_{t-2}
1.	$\hat{c}_3$	$\hat{c}_2$	$\hat{c}_1$
2.	$\hat{c}_4$	$\hat{c}_3$	$\hat{c}_2$
3.	$\hat{c}_5$	$\hat{c}_4$	$\hat{c}_3$
4.	$\hat{c}_6$	$\hat{c}_5$	$\hat{c}_4$
5.	$\hat{c}_7$	$\hat{c}_6$	$\hat{c}_5$
6.	$\hat{c}_8$	$\hat{c}_7$	$\hat{c}_6$
7.	$\hat{c}_9$	$\hat{c}_8$	$\hat{c}_7$
⋮	⋮	⋮	⋮
T-2.	$\hat{c}_T$	$\hat{c}_{T-1}$	$\hat{c}_{T-2}$

The first column contains the identifier of the observations, the second column refers to the LHS variable while the third and fourth columns refer to the lagged RHS variables. The indices within the table refer to actual time periods. The Granger-causality tests are calculated by practically running a regression with the rows of the table taken as independent observations. Now assume, that a crisis is identified in periods 5 and 6. Then, we have to delete observations (rows) 3, 4, 5 and 6 from the sample as these contain the crisis in one of the LHS or RHS variables. However, observations 1 and 2 still keep the appropriate lag structure while they does not contain the crisis periods. The same is true for observations 7 and beyond.

As a result, when controlling for the crisis periods we run the regressions in equations (1) and (2) with the crisis periods excluded as in the previous description. In other terms, we omit these periods from the regressions as we do with outliers in cross-sectional regression analysis.

Moreover, the HP-filter used to construct the business cycles naturally creates 'swings' around crisis periods: we observe positive output gaps before the crisis and negative ones during and after. In the case of a global crisis this leads to co-movements in all filtered time-series as output gaps synchronize

because of the filtering method. In order to control for this, we do not only exclude the periods after the beginning of the crisis, but also before the crises to rule out this kind of co-movements arising from the construction of the HP-filter. Our rule-of-thumb in this respect is to exclude the same amount of quarters before the crisis as the logic described above for the post-crisis quarters requires. In the example given in the table above, we omit 5 after-crisis period, so we also exclude 5 periods before the crisis (given that the length of the time window allows).

6. Network measures/topology:

Density

We used the graph.density function in the igraph package in R software to calculate the density of the networks. In order to use this function, we need to generate graphs from the adjacency matrices. So first we used the graph_from_adjacency_matrix function in igraph package to generate the unweighted, directed graphs.

Source: Csardi G, Nepusz T (2006). "The igraph software package for complex network research." InterJournal, Complex Systems, 1695. http://igraph.org.

Transitivity/clustering

We used the ClustBCG function in DirectedClustering packaege in R software to calculate the clustering coefficients of the networks. It computes local and global clustering coefficients for networks. For directed networks Clemente and Grassi formula is computed.

Source: Clemente, G.P. and Grassi, R. (2018) Directed clustering in weighted networks: a new perspective, Chaos, Solitons and Fractals, 107,26–38.

Modularity

For modularity, we need to symmetrize the adjacency matrices. So we used the same graph_from_adjacency_matrix function to create an unweighted, and undirected graph. We set the mode argument to "plus". In this case an undirected graph will be created with A(i,j)+A(j,i) edges between vertex i and j.

We used the modularity function in igraph package in R software to calculate how modular is the graph. For the *membership* argument we used the membership function from the same package. For the *communities* argument we used the cluster-louvain function in the same package. It is finding the community structure that maximizes the modularity indicator.

Average path length

To determine the average path length of the networks we used the average.path.length function in the igraph package in R. For this we used the created graphs. We set the *unconnected* argument to FALSE. In this case the length of the missing paths are counted having length vcount(graph), one longer than the longest possible geodesic in the network.

Skewness of degree

We calculated the degrees of the nodes of the graphs with the degree function in igraph package in R.

We used the skewness function in the e1071 package in R software, and we used the type=1 setting to calculate the skewness of the degrees in the networks.

7. Generating random networks as a reference network

We generated 1000 directed random graphs corresponding to every single time window according to the Erdős-Rényi model, using the erdos.renyi.game function in igraph in R. The probability for drawing an edge between two vertices is set as the density of the observed contagion network for the same time window.

We calculated all topological indicators for the 1000 random graph separately, then for every indicator we calculated the avarage of the 1000 indicators together with the 5 and 95 percentiles in order to have a reference range for them under the random network hypothesis.

8. Pairwise stability

When calculating stability, we used the relative frequencies of four different transitions in the network. Given the adjacency matrices A(i,j,t) for all time windows, for all time-switch (t,t+1) we identify the a country-pair as belonging one of the four transitions:

(1) no connection in t -> no connection in t+1
(2) no connection in t -> connection in t+1
(3) connection in t -> no connection in t+1
(4) connection in t -> connection in t+1

Then, using the cardinality of the four groups N_1, N_2, N_3, N_4, we calculate the relative frequency of each transition: $f_1,= N_1/(N^2-N)$ and so on, where $N^2-N = N_1+ N_2+ N_3+N_4$ is the total number of (directed) country pairs.

From the frequencies f, we can also calculate the transition probabilities as

$p_1 = f_1/(f_1+f_2)$

$p_2 = f_2/(f_1+f_2)$

$p_3 = f_3/(f_3+f_4)$

$p_4 = f_4/(f_3+f_4)$

9. Systematic contagion paths

Given the longitudinal nature of our data, we can extract pairwise time series from the adjacency matrices `A(i,j,t)`. Fixing i and j, we get a sequence of zeros and ones indicating whether we estimated shock contagion between from country i to country j in the subsequent periods.

On every single sequence of pairwise contagion histories we can run a Wald-Wolfowitz runs test in order to test the sequence composition against randomness, given the frequency of ones in the sample. For this, we used runstest.m in Matlab which provides as a result the logical result of the Hypothesis test that the given sequence is non-random (true) or random (false).

```
[H(i,j)] = runstest(A(i,j,:));
```

Given the matrix `H(i,j)` of logical values, we merge it with a density matrix `D(i,j)` which contains 1 if the given pair of countries had more observed contagion events (as estimated by Granger causality in time windows) than expected in 95% of random networks with equal density.

The final adjacency matrix plotted in panel A of Fig 4 was then constructed as

```
A(i,j) = H(i,j).*D(i,j).
```

The maximum spanning tree in panel B of Fig 4 was constructed on the basis of a directed, weighted version of the same graph. We used the original, estimated adjacency matrices `A(i,j,t)` again (with 0s and 1s in their entries) as a starting point. But instead of comprising the time dimension using the runs test, we simply sum the matrices along the time dimensions to get a weighted adjacency matrix, and symmetrize the matrix by summing bidirectional weights on a country-pair:

```
[F(i,j)] = sum(A,3);
```

Then, we employ the g_mst function in the igraph package with the Prim algorithm in R to obtain the minimal spanning tree of `1./F`, which corresponds to the maximum spanning tree of `F`.
