# Supplementary material for: Do economic shocks spread randomly?: A topological study of the global contagion network
Source: PLoS One. 2020 Sep 4;15(9):e0238626. doi: 10.1371/journal.pone.0238626 (PMC7473515; doi:10.1371/journal.pone.0238626)
Supplement: S1 Fig — These figures replicate Figs 1, 2, 3 and 4 with the configuration model as the randomized reference network. Also, Fig 5 is replicated with openness values restricted to trade within the sample countries. (PDF) [file pone.0238626.s005.pdf]

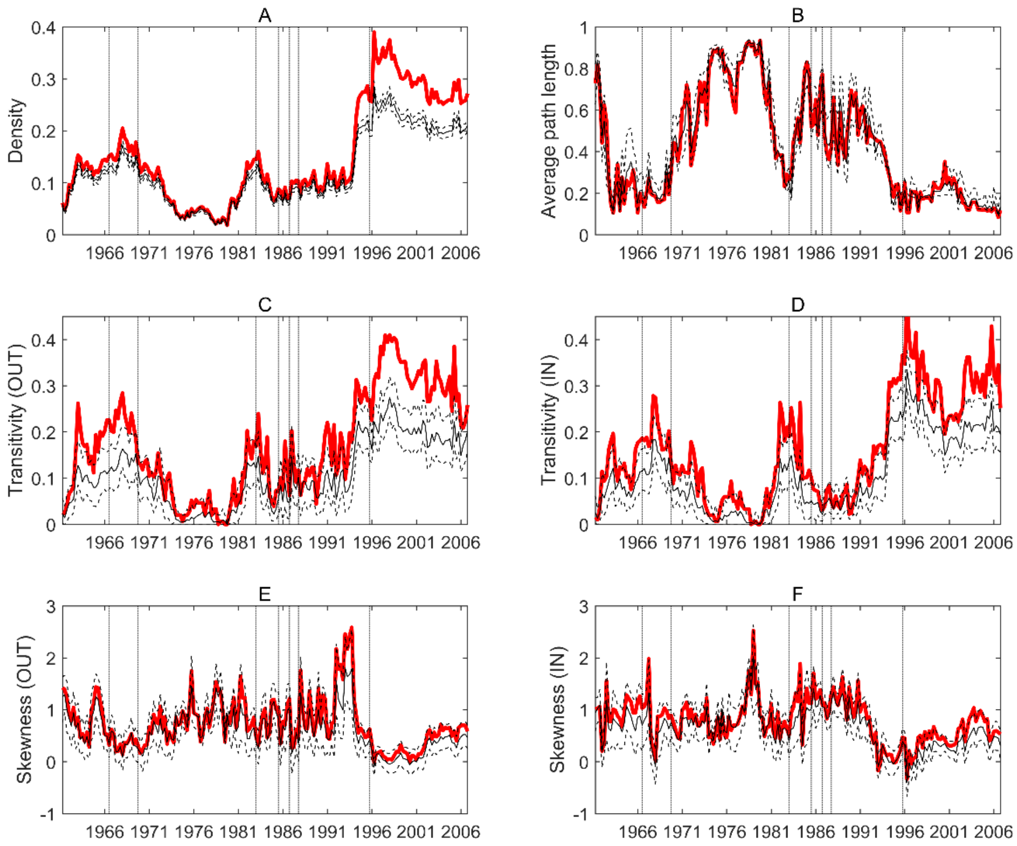


**Fig 1A. Topological characteristics of the global economic contagion network with CM graph as reference.** Every panel shows the evolution of a topological property. Red lines mark the observed values, solid black lines mark the average from simulated configuration model networks while dashed lines represent the 5 and 95 percentiles of the latter. Horizontal axes show time windows with the labels marking the initial year of the given window (e.g. the last label refers to 2006 which means that this data point represent the topological property of the contagion network estimated on the 52 quarters starting from 2006Q1. Vertical dotted lines mark those time windows in which a given crises first appears. A: Density. B: Average path length. C: Transitivity (transmission). D: Transitivity (exposure). E: Skewness (transmission). F: Skewness (exposure).}

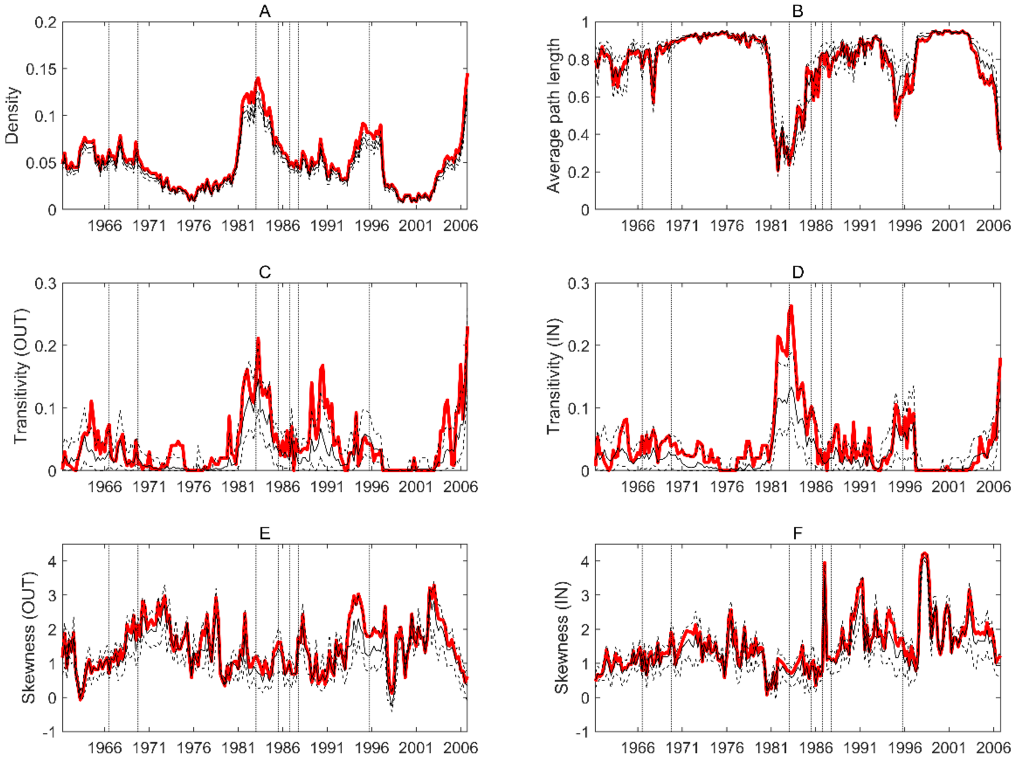


**Fig 2A. Topological characteristics of the global economic contagion network with crisis periods excluded, CM graph as reference.** Every panel shows the evolution of a topological property. Red lines mark the observed values, solid black lines mark the average from simulated configuration model networks while dashed lines represent the 5 and 95 percentiles of the latter. Horizontal axes show time windows with the labels marking the initial year of the given window (e.g. the last label refers to 2006 which means that this data point represent the topological property of the contagion network estimated on the 52 quarters starting from 2006Q1. Vertical dotted lines mark those time windows in which a given crises first appears. A: Density. B: Average path length. C: Transitivity (transmission). D: Transitivity (exposure). E: Skewness (transmission). F: Skewness (exposure).

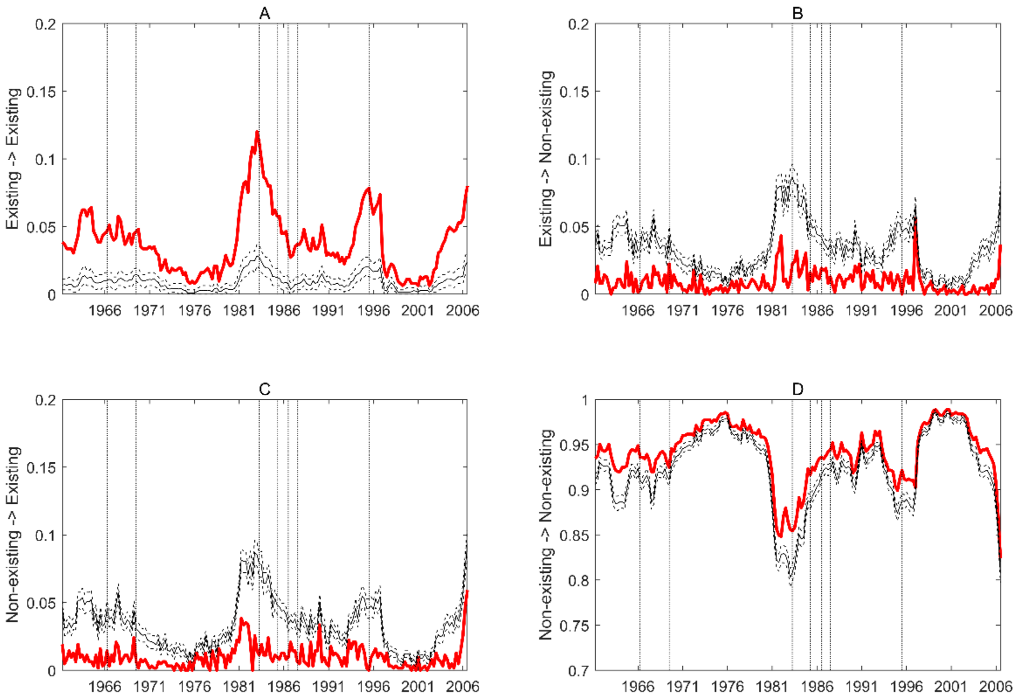


**Fig 3A. Link formation dynamics in the global economic contagion network, with CM graph as reference.** Every panel shows the path of a state transition frequency, where states refer to whether a connection exists or not. Red lines mark the observed values, solid black lines mark the average from simulated configuration model networks while dashed lines represent the 5 and 95 percentiles of the latter. Horizontal axes show time windows with the labels marking the initial year of the given window (e.g. the last label refers to 2006 which means that this data point represent the topological property of the contagion network estimated on the 52 quarters starting from 2006Q1. Vertical dotted lines mark those time windows in which a given crises first appears. A: Frequency of stable existing connections. B: Frequency of dissolving connections. C: Frequency of newly formed connections. D: Frequency of stable non-existing connections.

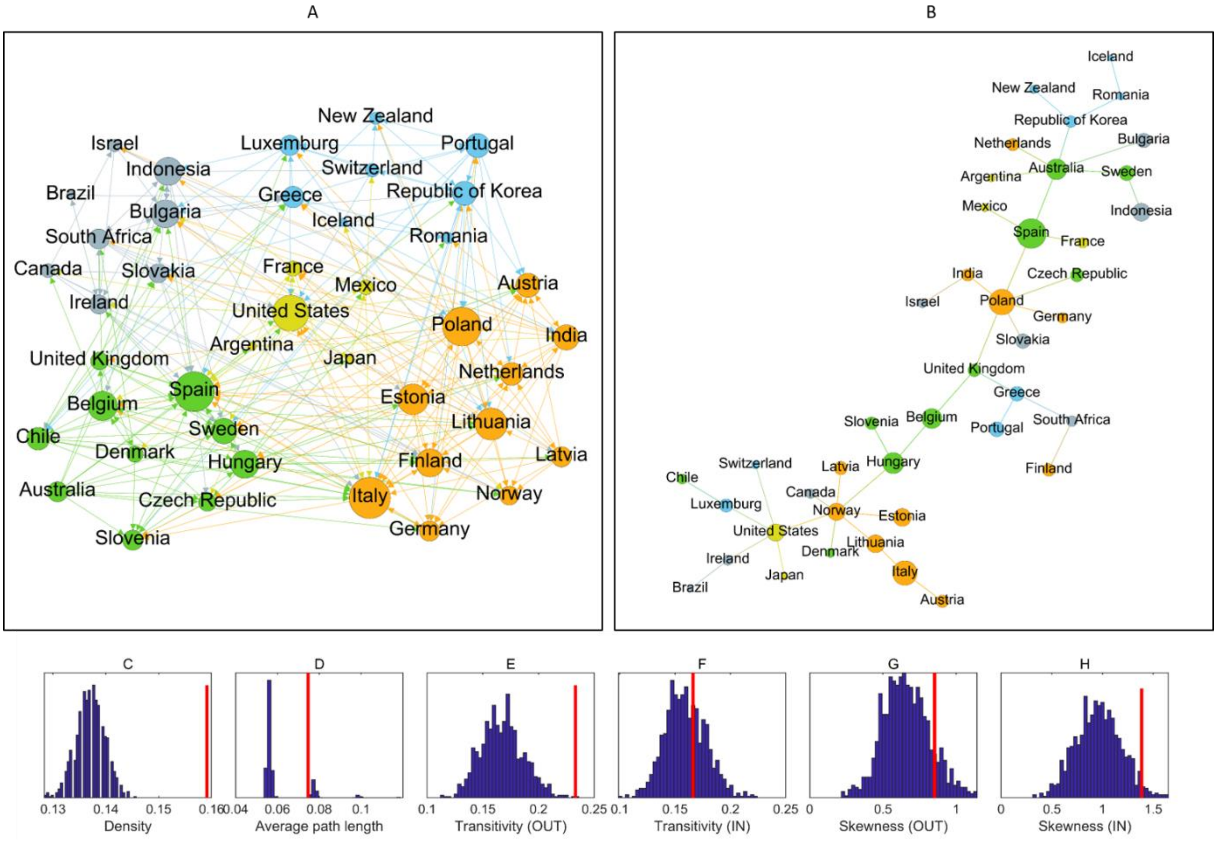


**Fig 4A. Systematic shock transmission paths in the global economic contagion network, with CM graph as reference.** Panel A shows the full network of systematically existing contagion paths. Node sizes reflect the sum of in- and outdegrees. Panel B shows the maximum spanning tree (MST) based on the systematically existing contagion paths. Coloring of the nodes in panels A and B reflect the modules. In panels C-H vertical red lines show observed topological properties in the mapped network while the blue histograms show the distribution of the same properties from a sample of 1000 independent configuration model networks. C: Density. D: Average path length. E: Transitivity (transmission). F: Transitivity (exposure). G: Skewness (transmission). H: Skewness (exposure).

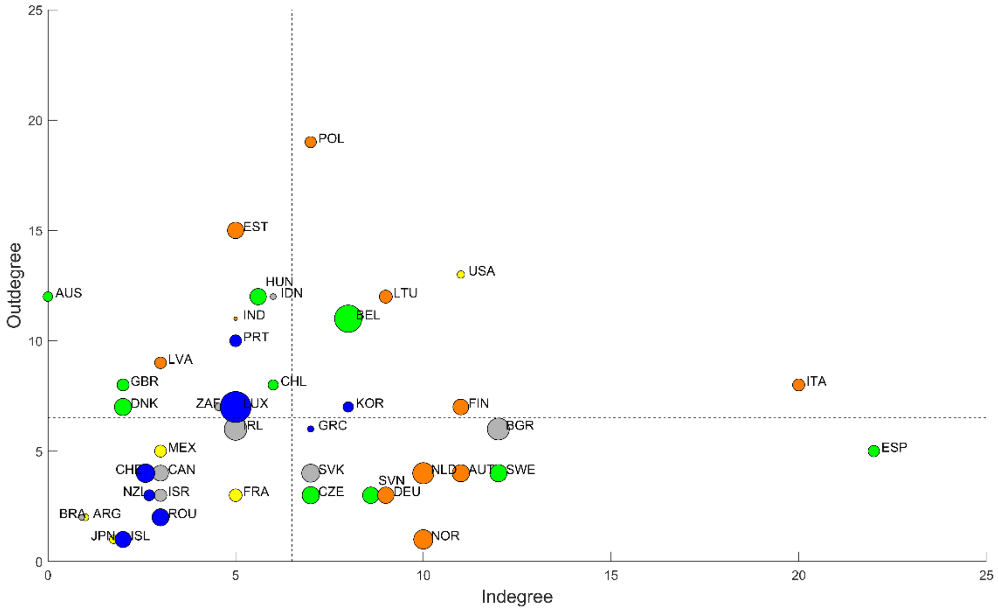


**Fig 5A. Within sample trade openness and connectivity.** Bubbles represent countries. Horizontal axis measures indegree in the contagion network depicted in panel A of Fig 4, vertical axis measures outdegree in the same network. The size of the bubbles are proportional to economic openness measured by within sample exports plus imports as a share of GDP, averaged over the period between 1996 and 2019 to match the time span of the network. Coloring reflects the modules, the dashed lines mark average in- and outdegrees.
